# A New Species of *Andesipolis* Whitfield & Choi from the Eastern Andes of Ecuador with Notes on Biology and Classification (Hymenoptera: Braconidae: Rhysipolinae)

**DOI:** 10.1673/031.009.3601

**Published:** 2009-06-02

**Authors:** Andrew C. Townsend, Scott R. Shaw

**Affiliations:** ^1^ Yanayacu Biological Station, Napo prov., Cosanga, Ecuador; ^2^ Department of Renewable Resources, University of Wyoming

**Keywords:** Mesostoinae, Pyralidae, *Phenax rugosus*, *Boehmeria bullata*

## Abstract

A new species of braconid wasp, *Andesipolis yanayacu*, is described from the eastern Andes of Ecuador. *Andesipolis yanayacu* was reared as a gregarious koinobiont parasitoid of shelter building Pyralidae (Lepidoptera) larvae feeding on Urticaceae (*Phenax rugosus* and *Boehmeria bullata*). These are the first biological observations for the genus *Andesipolis* and the first species recorded from Ecuador. This is also the northern-most record for the genus as previously described species are from Chile. Based on morphological attributes and the newly discovered biology, *Andesipolis* is re-classified from the subfamily Mesostoinae into the subfamily Rhysipolinae.

## Introduction

The “cyclostome” braconids, so called because their labrum and clypeus are indented to form a characteristic concave depression, have been, and continue to be problematic with regards to classification (Hedqvist 1963; Shaw 1983; Shaw and Huddleston 1991; Quicke 1993; Wharton 1993; Achterberg 1995; Whitfield and Wharton 1997; Quicke and Belshaw 1999; Spencer and Whitfield 1999; Whitfield et al. 2004; Shi et al. 2005; Zaldivar-Riveron et al. 2006). Various classifications of subfamilies, tribes and genera exist for cyclostome braconids (see Hedqvist 1963; Wharton 1993; Achterberg 1995; Shaw 1995; Wharton et al. 1997; Whitfield and Wharton 1997), but there is a general lack of consensus. This, coupled with the fact that some cyclostome genera fall outside current definitions of subfamilies and tribes, has led to problems in classification (Whitfield and Wharton 1997; Whitfield et al. 2004).

Even considering these major classification problems, as well as the likelihood that changes will take place when a complete overhaul of the group is undertaken, it remains useful to suggest taxonomic placement of newly described species. The new species of *Andesipolis* Whitfield and Choi (2004), *A. yanayacu*, described here, is a case in point.

In their description of *Andesipolis*, Whitfield et al. (2004) did not attempt to place the genus unambiguously into a tribe or subfamily due to its unique nature and the above-mentioned problems. They instead outlined similarities of the genus with different groups including Rhysipolinae and Rhyssalinae. However, in a recent molecular and morphological analysis of the systematics of cyclostome braconids, Zaldivar-Riveron et al. (2006) placed *Andesipolis* within the small and rare subfamily Mesostoinae. This is an intriguing proposal, but our examination of *Andesipolis* morphology and newly discovered biology supports placement within the Rhysipolinae.

There are only three described species of *Andesipolis*, although additional undescribed species represented by single specimens or males only are known. *Andesipolis*, up until discovery of *A. yanayacu*, has only been found in the Andean region of Chile and the biology was unknown (Whitfield et al. 2004).

## Materials and Methods

All of the specimens for this study were reared as part of the *Caterpillars and Parasitoids of the Eastern Andes of Ecuador* project (NSF-BSI-0346729; NSF-BSI-0717458; Dyer et al., 2008) at the Yanayacu Biological Station, Napo province. Larval Lepidoptera were sampled by walking through various habitats and inspecting herbs, shrubs and trees up to a height of approximately 2.5 m. Caterpillars were collected in clear plastic bags along with their food plant and transported to a rearing shed at the research station. Caterpillars collected were assigned an identification code and their identity and food plant were recorded. Rearing took place in plastic bags in an open air, covered shelter where the caterpillars were exposed to ambient temperatures and day length, and fed natural food plants from the field. Frass and unused or decaying plant material were removed every other day whereupon new plant material was provided as necessary. While cleaning out the bags, the caterpillars were also inspected to note date of caterpillar pupation or date of parasitoid pupation. Parasitoid pupae were inspected daily for emergence. Parasitoid specimens were maintained with the original code given to the caterpillar upon collection to preserve all host and host plant data recorded. Parasitoids were preserved in alcohol and transferred to the University of Wyoming where they were dried and point mounted on pins for identification.

Specimens of *Andesipolis* can be keyed to genus using the key to “hormiine” genera (Whitfield and Wharton 1997) in the *Manual of the New World Genera of the Family Braconidae* (Wharton et al. 1997). The existence of the genus was known at the time of publication of the manual, but it was not formally described. Therefore, *Andesipolis* specimens appear in the key as “undescribed genus 2” (Whitfield and Wharton 1997; Whitfield et al. 2004). For a diagnosis of the genus as well as photos of diagnostic features see Whitfield et al. (2004).

Terminology follows that used for *Andesipolis* by Whitfield et al. (2004). Microsculpture terminology follows Harris (1979). Wing vein terminology follows the system of Sharkey and Wharton (1997).

Body length was measured dorsally as head and mesosoma plus metasoma. Dorsal head width was measured including eyes. Dorsal thorax (mesosoma) width was measured at the widest point on the mesoscutum between the tegula. Mesonotum length was measured dorsally. Cocoon width was measured at mid-length. Scanning electron microscopy was done at the University of Wyoming Microscopy Core Facility using a Hitachi tabletop scanning electron microscope (SEM), model TM-1000. Specimens were examined uncoated at an operating voltage of 15 kV.

### Biology

The biology of *Andesipolis* is reported here for the first time (Whitfield et al. 2004); however, the biology of the Rhysipolinae, including the similar-looking genus *Rhysipolis*, is known. Rhysipolines are known to be solitary koinobiont ectoparasitoids of concealed Lepidoptera; larvae develop externally while the host continues to develop (van Achterberg 1995; Shaw 1995; Shaw and Huddleston 1991; Shaw 1983; Spencer and Whitfield 1999). *Rhysipolis* species attack leaf-mining and shelter-building Lepidoptera and spin cocoons on the undersides of leaves or within the shelters constructed by their hosts (Shaw 1983; and Spencer and Whitfield 1999). Because they possess a long ovipositor, Whitfield et al. (2004) predicted that members of *Andesipolis* attack hosts within leaf shelters, stem tissue, or plant galls.

**Figure 1.**
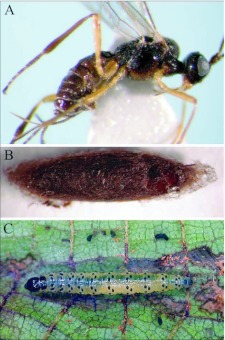
Images of *Andesipolis yanayacu* n sp. and host caterpillar. (A) Lateral habitus, length = 2.9 mm. (B) Cocoon, length = 3.4 mm. (C) Undetermined pyralid (Lepidoptera) host.

Ten of the twelve specimens of *Andesipolis yanayacu* examined in this study were reared from three individuals of a single shelter-building species of Pyralidae (Lepidoptera) (Figure 1C), supporting the prediction put forth by Whitfield et al. (2004). The two remaining specimens were reared from collected parasitoid cocoons (Figure 1B). No externally developing wasp larvae were noticed by eye; however field-collected caterpillars were not examined under magnification so ectoparasitism cannot be ruled out. Host development was not arrested after parasitism, indicating that *A. yanayacu* is a koinobiont. Caterpillars parasitized by *A. yanayacu* were collected in June, 2006 and adult parasitoids emerged in July, 2007. All individuals spun their own silken cocoon (Figure 1B) within a mass of other cocoons. All cocoons of a respective gregarious brood were loosely bound together by a fine silk mesh within the host shelter among frass.

Two of the host caterpillars fed on *Phenax rugosus* (Urticaceae), while the third host fed on *Boehmeria bullata* (Urticaceae). The caterpillars collected on *Phenax rugosus* produced gregarious broods of five and four parasitoid individuals with female to male sex ratios of 4:1 and 1:3 respectively. The caterpillar collected from *B. bullata* hosted several parasitoid individuals that spun cocoons from which only one male emerged. All wasp cocoons were spun within the leaf shelter constructed by the host, similar to the concealed pupation behavior seen in *Rhysipolis* (Shaw 1983; Shaw and Huddleston 1991; Spencer and Whitfield 1999).

## Descriptive taxonomy

**Figure 2.**
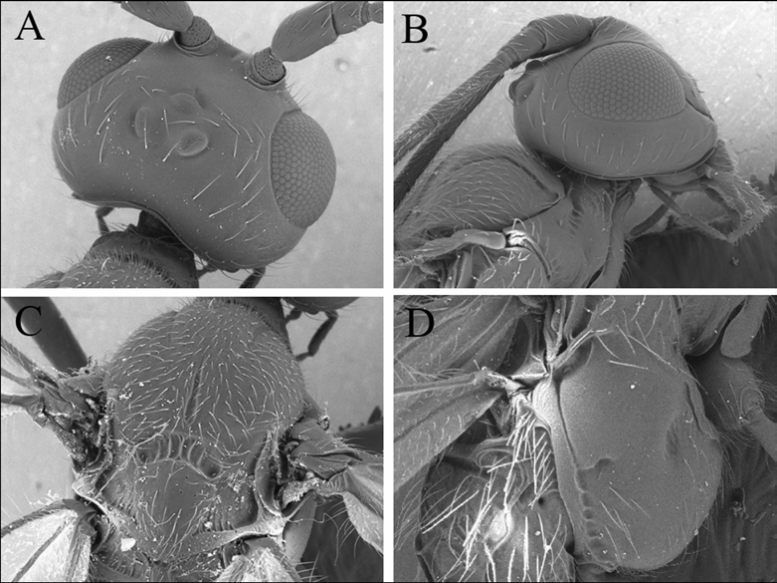
SEM images of *Andesipolis yanayacu* n sp. (A) Head and pronotum, dorsal aspect, 300x. (B) Head and pronotum, lateral aspect, 250x. (C) Mesonotum and scutellum, dorsal aspect, 250x. (D) Mesopleuron, lateral aspect, 300x.

**Figure 3.**
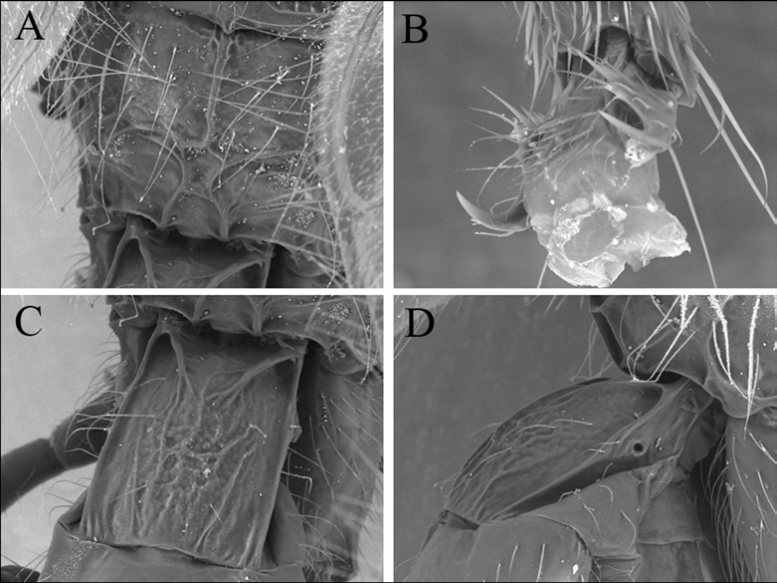
SEM images of *Andesipolis yanayacu* n sp. (A) Propodeum, dorsal aspect, 500x. (B) Tarsal claw, 1,800x. (C) Metasomal tergite 1, dorsal aspect, 500x. (D) Metasomal tergite 1, lateral aspect, 500x.

***Andesipolis yanayacu* Townsend and Shaw, new species (Figures 1–3)**
♀.***Body color:*** (Figure 1A) body mostly black; oral cavity, labium, maxillary palpus, and labial palpus honey yellow; mandible light brown; abdomen dark brown except for black tergite 1, ovipositor honey yellow; legs mostly honey yellow, apical 2 segments of fore and mid tarsus dark brown, hind tibia fading from light brown basally to dark brown apically, tarsus brown.
***Body length:*** = 2.9 mm; forewing length = 3.1 mm.
***Head:*** (Figures 1A, 2A, 2B) 1.40 × wider than mesoscutum, entirely smooth and polished with scattered setae; clypeus 2.4 × as broad as its height; malar space 1.5 × basal mandibular width; malar suture present; eye about 1.4 × higher than wide; occipital and hypostomal carina converging near mandibular base; antenna slightly longer than forewing, comprising 31 segments, first flagellomere roughly 4 × longer than wide, remaining flagellomeres all longer than wide with a progression from a higher length/width ratio basally to a lower length/width ratio apically; ocelli small, ocell-ocular distance about 3 × the width of lateral ocellus; maxillary and labial palpus 6-and 3-segmented respectively.
***Mesosoma:*** (Figures 1A, 2A, 2B, 2C, 2D, 3A) 2.25 × longer than width between tegula; pronotum mostly smooth, with irregular indentations; mesonotum punctate with scattered setae, notauli weak and incomplete, midpit shallow and elongate coming to a point apically, roughly 0.3 X the length of mesonotum; scutellar disc smooth with scattered setae, scutellar sulcus with longitudinal carina; mesopleuron mostly smooth and highly polished, sternaulus slightly rugose, epicnemial carina present; propodeum with areola, and areolar cross bridge, median carina present.
***Legs:*** (Figures 1A, 3B) fore coxa swollen and round, hind coxa smooth with scattered setae; tarsal claw simple.
***Wings:*** (forewing) stigma roughly 4.6 × longer than broad; vein r arising from middle of stigma; vein r slightly less than ⅓ length of vein 3RSa; vein 2RS 0.7 × as long as vein 3RSa; vein 3RSa 0.6 × as long as vein 3RSb; vein r-m 0.4 × the length of vein 3RSa; vein 1CUb 2.3 × as long as vein 1CUa; vein m-cu meets vein RS+M before RS splits from M; vein 2a spectral, arising slightly before vein 1cu-a. (Hindwing): vein M+CU 1.7 × length of vein 1M; vein cu-a 0.4 × length of vein 1M; vein r-m 0.6 × length of vein 1M; vein m-cu absent.
***Metasoma:*** (Figures 1A, 3C, 3D) tergite I heavily sclerotized and rugose, length 1.1 × apical width, dorsal carina converging anteriorally but not meeting, dorsope large; remainder of abdominal terga smooth and polished with scattered setae; spiracle of metasomal tergite II situated near lateral edge of dorsal face; ovipositor roughly equal to length of hind tibia, curved.
  ♂: Essentially as in ♀ except metasomal tergite 1 length 1.2 to 1.3 × apical width.
***Variation:*** Body length = 2.6–2.9 mm. Forewing length = 2.8–3.1 mm. Head width 1.3–1.4 × mesoscutum width; clypeus 2.2–2.6 × as broad as high; antennal segments 31–32; mesosoma 2.1–2.4 × longer than width; mesonotum sometimes rugose postero-medially, partly obscuring midpit; areola and areolar cross bridge faintly present to distinctly present.
***Holotype:*** ♀. Ecuador, Napo province, Yanayacu Biological Station, S 00°35.9′, W 77°53.4′, 2163m, 3-July-2006, reared, host plant, *Phenax rugosus*, host, Pyralidae. Deposited in University of Wyoming Insect Museum (UWIM).
***Paratypes:*** 3 ♂♂, 3 ♀♀, same data as holotype with dates ranging from 3-July-2006 to 7-July-2006 (UWIM). 1 ♂, 1 ♀, same data as holotype with dates ranging from 3-July-2006 to 7-July-2007. Deposited in Museo Ecuatoriano de Ciencias Naturales (MECN). 1 ♂, Ecuador, Napo province, Yanayacu Biological Station, S 00°35.9′, W 77°53.4′, 2163m, 1-July-2006, reared, host plant, *Boehmeria bullata*, host, Pyralidae. Deposited in British Museum of Natural History (BMNH). 1 ♂, 1 ♀, Ecuador, Napo province, Yanayacu Biological Station, S 00°35.9′, W 77°53.4′, 2163m, 2-April-2005, collected as cocoons and reared. Deposited in U.S. National Museum of Natural History (USNM).

### Distribution

Known only from the type series collected on the eastern slope of the Andes in Napo, northeastern Ecuador.

### Biology

Reared as a gregarious koinobiont from an undetermined shelter-building Pyralidae (Lepidoptera) (Figure 1C), feeding on *Phenax rugosus*, and *Boehmeria bullata* (Urticaceae).

### Cocoon

Dark brown, tightly woven silk layer surrounded by a somewhat wooly outer silk layer, ovoid; length 3.3–3.9 mm, width 1.1–1.2 mm; exit hole irregular (Figure 1B); multiple cocoons loosely bound together by a fine silk mesh within the host shelter among frass.

### Comments

Currently there is only three described species of *Andesipolis*. This species differs from *Andesipolis masoni* Choi & Suh and *A. framea* Whitfield & Choi by the presence of a curved ovipositor (Figure 1A), and differs from *A. whartoni* Whitfield & Choi by having simple tarsal claws without a basal tooth (Figure 3B).

### Etymology

The species name refers to the rearing locality at the Yanayacu Biological Station in Napo Province, Ecuador.

## Discussion

Zaldivar-Riveron et al. (2006), based on morphological and molecular data, have suggested placement of *Andesipolis* within the Mesostoinae. Superficially, *Andesipolis* specimens resemble mesostoines by the presence of a hypoclypeal depression, and largely smooth and weakly sclerotized metasomal segments beyond segment one. However, *Andesipolis* specimens lack many diagnostic features of the Mesostoinae including: strong reduction of maxillary and labial palpi, a mesoscutum that protrudes above the pronotum, strongly compressed legs (especially femora), fewer than 30 antennal segments, and absence of the epicnemial carina (Quicke and Huddleston 1989). Furthermore, all mesostoines for which life histories are known are phytophagous gall formers (Austin and Dangerfield 1998). We discovered that *Andesipolis yanayacu* is a koinobiont parasitoid of shelter-building Lepidoptera. This argues against the placement of *Andesipolis* in the Mesostoinae.

In their work on the systematics of cyclostomes, Zaldivar-Riveron et al. (2006: p. 140) stated “our molecular and morphological evidence clearly shows that the aberrant South American genus *Andesipolis* belongs to the Mesostoinae.” However, examination of the supplementary data from their study reveals that some of the morphological characters used in the analysis were incorrectly scored for both *Rhysipolis* and *Andesipolis*. For example, *Rhysipolis* was scored as not having a hypoclypeal depression, even though in the only *Rhysipolis* revision done (Spencer and Whitfield 1999), a diagnostic feature of the genus is the presence of the labrum within an oval hypoclypeal depression. Furthermore, *Rhysipolis* was scored by Zaldivar-Riveron et al. (2006) as having hind wing vein m-cu present, whereas a diagnostic feature of the subfamily Rhysipolinae is the absence of an m-cu vein (Achterberg 1995). Similarly, *Andesipolis* was scored as having the occipital carina meeting the hypostomal carina before reaching the base of the mandible, while the previously described Chilean *Andesipolis* species exhibit another pattern: the two carinae remain separate to the base of the mandible (Whitfield et al. 2004). These coding choices may have affected the outcome of the Zaldivar-Riveron et al. (2006) results.

We propose an alternative solution: *Andesipolis* specimens resemble rhysipolines in many morphological traits including: presence of a malar suture; the dorsal carina of tergite I converging; forewing vein m-cu joining RS+M before RS splits from M; absence of hind wing vein mcu; the hind tarsus being roughly equal in length to the hind tibia; and a long median carina on the propodeum. In addition, *Rhysipolis* species are known to attack leafmining and shelter-building Lepidoptera, spinning their cocoons on the undersides of leaves or within the shelters constructed by their hosts (Shaw 1983; Spencer and Whitfield 1999). Similar host association, as well as pupation behavior, was observed in *A. yanayacu*. These morphological and biological characteristics suggest that *Andesipolis* is better placed within the Rhysipolinae than within the Mesostoinae.

### Editor's note

Paper copies of this article will be deposited in the following libraries. Senckenberg Library, Frankfurt Germany; National Museum of Natural History, Paris, France; Field Museum of Natural History, Chicago, Illinois USA; the University of Wisconsin, Madison, USA; the University of Arizona, Tucson, Arizona USA; Smithsonian Institution Libraries, Washington D.C. USA; The Linnean Society, London, England.
